# E-Cigarette Vapour Alters High-Fat Diet-Induced Systemic Inflammatory Responses but Has No Effect on High-Fat Diet-Induced Changes in Gut Microbiota

**DOI:** 10.3390/nu15071783

**Published:** 2023-04-06

**Authors:** Hui Chen, Catherine Burke, Chantal Donovan, Alen Faiz, Sonia Saad, Brian G. Oliver

**Affiliations:** 1 School of Life Sciences, Faculty of Science, University of Technology Sydney, Sydney, NSW 2007, Australiabrian.oliver@uts.edu.au (B.G.O.); 2 Hunter Medical Research Institute, The University of Newcastle, Callaghan, NSW 2308, Australia; 3 Renal Group, Kolling Institute of Medical Research, The University of Sydney, St Leonards, NSW 2064, Australia; 4 Respiratory Cellular and Molecular Biology, Woolcock Institute of Medical Research, Macquarie University, Glebe, NSW 2037, Australia

**Keywords:** e-cigarettes, e-vapour, high-fat diet, microbiome, gut microbiota, lipids

## Abstract

Background: The gut microbiome, which can be altered by different diets or smoking, has been implicated in the pathogenesis of lung conditions. E-cigarette vaping is now recognised to have detrimental health effects, with several of these being similar to cigarette smoking. However, whether e-cigarettes can alter high-fat diet (HFD)-induced systemic effects and gut microbiota is unknown. In this study, we investigated the effects of HFD in the absence/presence of e-cigarette exposure on systemic inflammation, lipid metabolic markers, and the gut microbiome. Methods: Mice were fed a HFD (or chow) in the absence/presence of e-vapour exposure (±nicotine) and serum inflammation, lipid levels, and microbial diversity were assessed. Results: HFD increased the circulating levels of both triglycerides and non-esterified fatty acids, which were significantly reduced by e-vapour exposure in HFD-fed mice. Serum TNF-α was increased by HFD consumption or e-vapour. HFD had a significant effect on microbial diversity, but there were no additional effects of e-vapour exposure. Conclusions: This study highlights both similarities and differences in how the body responds to e-cigarette vapours, and it is therefore likely that the long-term sequelae of e-cigarette vapour exposure/vaping might not involve the significant alteration of the gut microbiome.

## 1. Introduction

The gut microbiota has been implicated in the pathogenesis of lung disorders, and recent studies suggest that bidirectional crosstalk between the gut and the lung (known as the gut–lung axis) may influence the development of certain lung diseases [1]. Furthermore, smoking is well known to alter gut microbiota, leading to the development of various chronic diseases [2]. Comparatively, little is known about the long-term health effects of e-cigarette use (e-vapour exposure, both in the absence or presence of nicotine) on the diversity of gut microbiota, although in vivo models and human studies on the occurrence of acute lung injury and related mortality have highlighted detrimental health effects similar to cigarette smoking [3].

Diet is the most prominent factor in changing gut microbiota, and poor diets are closely related to the development of obesity and metabolic disorders [4]. Interestingly, a population-based study suggested that e-cigarettes are preferred over cigarettes by overweight and obese people [5]. However, the effects of a combined long-term high-fat diet (HFD) and e-cigarette use on the diversity of gut microbiota and its correlation with metabolic disorders and systemic inflammatory response are less well understood, given the more recent appearance of e-cigarettes on the market. Therefore, in this study, we modelled the effects of long-term HFD consumption in the absence or presence of e-vapour (±nicotine) in a mouse model to study the changes in body weight, metabolic markers, systemic inflammation, and gut microbiota to investigate the hypothesis that e-vapour exposure can change microbiota in a nicotine-dependent manner.

## 2. Materials and Methods

### 2.1. In Vivo Model

**Figure 1:**
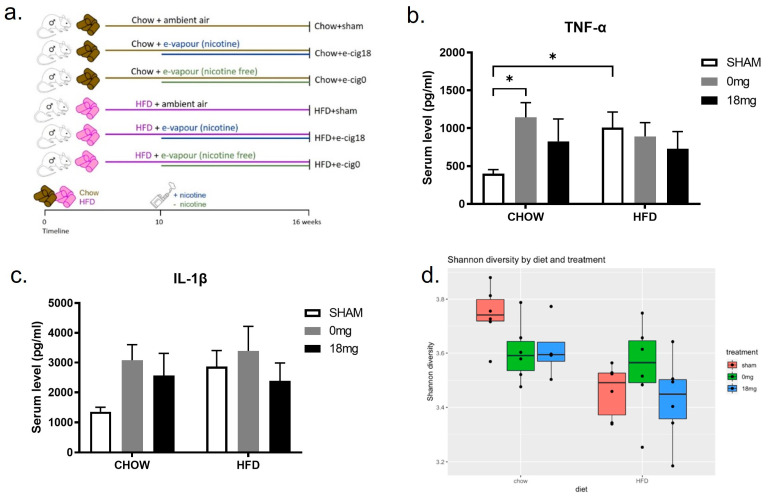
Effects of HFD- or chow-fed mice in the absence or presence of e-cigarette exposure (±nicotine). (**a**) Treatment timeline and study groups. Serum levels of proinflammatory cytokines (**b**) TNF-α and (**c**) IL-1β. (**d**) Alpha diversity analysed in faeces. Results are expressed as mean ± S.E.M, *n* = 5–6. * *p* < 0.05. e-cig18: exposed to nicotine-containing e-vapour; e-cig0: exposed to nicotine-free e-vapour; HFD: high-fat diet; sham: exposed to ambient air.

This study was approved by the Animal Ethics and Care Committee at Northern Sydney Health District (RESP17/93). All experiments were performed according to the Australian National Health & Medical Research Council (NHMRC) Guide for the Care and Use of Laboratory Animals. Balb/c mice (7 weeks old) were fed a HFD (43% fat, 20 kJ/g, Specialty Feeds, Glen Forrest, WA, Australia) for 10 weeks to induce obesity or standard chow as the control (14% fat, 14 kJ/g, Gordon’s Specialty Stock Feeds, Yanderra, NSW, Australia). From weeks 11–16, two sub-groups of mice (*n* = 6) in each dietary group were exposed to nicotine-containing e-vapour (18 mg/mL nicotine, tobacco flavour, 50% Propylene Glycol [PG]/50% Vegetable Glycerine [VG], Vaper Empire, VIC) or nicotine-free e-vapour (0 mg/mL, tobacco flavour, 50% PG/50% VG, Vaper Empire, VIC) for 30 min twice daily for 6 weeks using previously published protocols [6,7,8], while the same diets were maintained. This resulted in 6 groups: Chow + sham, Chow + e-cig18, Chow + e-cig0, HFD + sham, HFD + e-cig18, and HFD + e-cig0 (Figure 1a).

Mice started with similar body weights across all groups. Body weight was measured weekly while the animals were conscious and at the endpoint after deep anaesthesia (2% isoflurane). Retroperitoneal and epididymal fat pads were dissected and weighed. Blood was collected via cardiac puncture at the endpoint, and serum samples were kept at −20 °C. A Bio-Plex Pro™ Mouse Chemokine Panel 33-Plex kit (Bio-Rad, Hercules, CA, USA) was used to measure TNF-α and IL-1β levels in the serum following the manufacturer’s instructions. Serum non-esterified free fatty acids (NEFA) were measured using a commercial NEFA kit (WAKO, Osaka, Japan) following the manufacturer’s instructions. Serum triglycerides were measured by an in-house assay using glycerol standards (Sigma-Aldrich, St. Louis, MO, USA) and triacylglycerol reagent (Roche Diagnostics, Basel, Switzerland) [9].

### 2.2. Microbiome Analysis

One faecal pellet from each mouse at the endpoint was processed for DNA analysis using MoBio PowerFecal DNA extraction kits, as per the manufacturers’ instructions. DNA was quantified via Nanodrop. The V3–V4 region of the 16S rRNA gene was amplified from faecal DNA using the Zymo Quick-16S NGS Library Prep kit, as per the manufacturers’ instructions, and sequenced on a MiSeq V3 flow cell with 2 × 300 paired-end reads. Data were processed using the Qiime2 (version 2019.10) [10] and the implementation of the following programs: read pairs were merged using vsearch [11], merged sequences were then trimmed to remove primers, and deblur [12] was applied to generate amplicon sequence variants (ASVs) and a feature table of counts of ASVs per sample. A naïve bayes classifier was trained on the V3-V4 region and used to classify ASVs taxonomically using Scikit Learn [13]. A phylogenetic tree was generated using Fasttree2 [14], and ANCOM [15] was applied to determine significant differences in ASVs between groups. Downstream analyses were performed in R (https://www.R-project.org/ (accessed on 27 August 2021)). Shannon diversity and UniFrac distances and ordination were calculated using the Phyloseq package [16] and TidyR (Tidy Messy Data. R package version 1.0.0, https://CRAN.R-project.org/package=tidyr (accessed on 27 August 2021)) and ggplot2 [17] were used to handle data and generate plots.

### 2.3. Statistical Analyses

All data are presented as means ± S.E.M. Comparisons between multiple groups were performed using a two-way ANOVA with a post hoc Fisher’s least significant difference test. Statistical analyses were performed using GraphPad Prism software v9 (GraphPad Software Inc., San Diego, CA, USA).

## 3. Results

### 3.1. E-Cigarette Exposure Reduces High-Fat Diet-Induced Increases in Retroperitoneal Fat, Serum Triglycerides, and Non-Esterified Fatty Acids

HFD consumption increased body weight and fat mass compared to control chow (Table 1). In chow-fed mice with e-vapour exposure, there was a trend to reduce body weight and fat mass, whereas these parameters were significantly reduced in HFD-fed mice independent of the presence of nicotine (*p* < 0.05 e-vapour exposure effect, Table 1). HFD consumption also significantly increased circulating lipid levels, including both triglycerides and NEFA (*p* < 0.01 HFD effect, Table 1), which were significantly reduced by e-vapour exposure in HFD-fed mice. In chow-fed mice, NEFA levels were reduced by nicotine-containing e-cigarettes (Table 1). Serum TNF-α was increased through HFD consumption and exposure to nicotine free e-vapour (Figure 1b), whilst IL-1β was not changed (Figure 1c). Together, these data show that e-vapour exposure can reduce HFD-induced increases in retroperitoneal fat, serum triglycerides, and NEFAs, and increase TNF-α in the absence of HFD.

### 3.2. E-Cigarette Exposure Has No Effect on HFD-Caused Reduction in Microbiome Diversity

**Figure 2:**
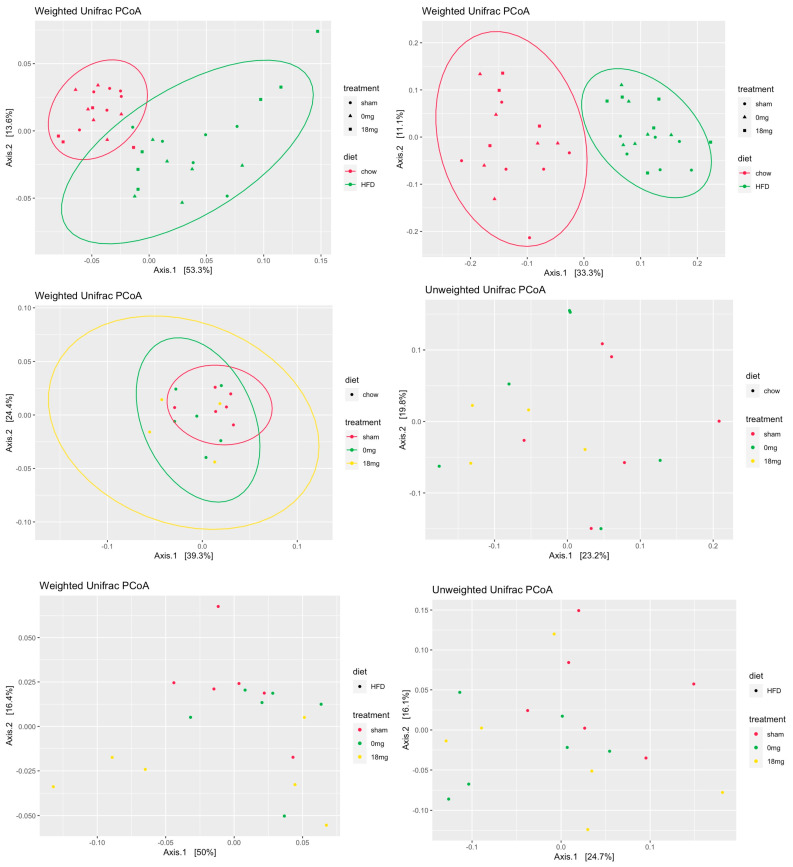
Beta diversity. HFD: high-fat diet; sham: exposed to ambient air; 18 mg: exposed to nicotine-containing e-vapour; 0 mg: exposed to nicotine-free e-vapour.

For all measures of changes in the microbiome (alpha and beta diversity), the same effect was observed. HFD had a strong and significant effect on diversity, but there was no significant effect of e-vapour exposure in the chow or HFD-fed groups (Figure 1d). E-vapour exposure showed a significant effect in the chow-fed mice on the unweighted UniFrac distance, meaning that there are certain operational taxonomic unit (OUT) differences for presence/absence between treatments (Figure 2). The weighed UniFrac distance was not significantly different, suggesting that these differences occur in OTUs of low relative abundance. Both for the weighted and unweighted analyses, HFD had a strong effect on the structure of the microbial community (Figure 2).

E-vapour exposure did not show a significant effect on microbiome diversity. There was a significant effect of e-vapour exposure on unweighted UniFrac only in the chow-fed mice. Together, these data suggest that there may be some subtle differences in terms of which microbes are present or absent, but when relative abundance is taken into account, these differences are not significant.

**Figure 3:**
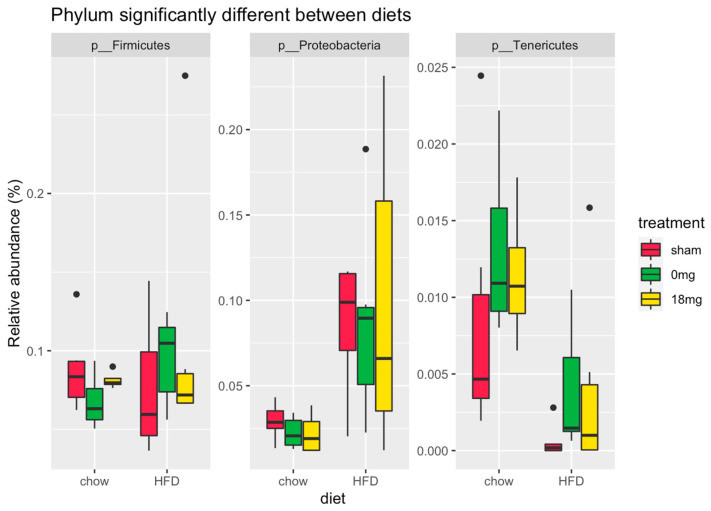
Abundance by diet. HFD: high-fat diet; sham: exposed to ambient air; 18 mg: exposed to nicotine-containing e-vapour; 0 mg: exposed to nicotine-free e-vapour.

HFD increased the relative abundance of the phyla Firmicutes and Proteobacteria and decreased the relative abundance of Tenericutes (Figure 3). The differences due to treatment were much more subtle (<0.1% difference in relative abundance and for taxa present only at very low relative abundance (<0.5% relative abundance)) (Figure 3).

## 4. Discussion

In this study, we show that e-vapour exposure induced increases in circulating inflammatory markers and demonstrated toxicity as reflected by reduced body weight in both the chow and HFD-fed groups. Surprisingly, we found no additional effects of e-vapour exposure on HFD-induced changes in the gut microbiota.

A recent systemic review on the potential role/s of e-cigarettes/e-vapour and their effects on body weight [5] highlighted the importance of mechanistic studies into the role of e-cigarettes on weight change and lipid accumulation. This systemic review also confirmed the prevalence of e-cigarette use in obese or overweight individuals [5]. In our current study, we observed that e-vapour exposure reduced body weight in HFD-fed mice compared to chow-fed mice. In addition, there was a decrease in fat mass in HFD-fed mice exposed to e-vapour in the absence or presence of nicotine, compared to HFD-fed mice alone. These data align with the clinical observations of weight loss associated with e-cigarette exposure in obese and overweight individuals [5]. Furthermore, this systemic review also suggested that e-cigarette exposure may alter energy metabolism and cause the dysregulation of inflammatory cells and immune processes, which may result in reduced lipid accumulation. HFD consumption can directly impact intestinal epithelial cells through the increased release of proinflammatory cytokines and changes in the gut microbiota. This alteration in gut microbiota leads to the elevated production of lipopolysaccharides. The presence of endotoxins and proinflammatory cytokines in the bloodstream contributes to a state of low-grade systemic inflammation, leading to the disruption of organ homeostasis. Indeed, we observed an HFD-dominant impact on the gut microbiome to reduce alpha diversity. Interestingly, we found that HFD-fed mice exposed to e-vapour had reduced circulating lipid levels compared to sham-exposed mice fed an HFD, suggesting that nutrient metabolism and immune cell responses may also be altered in our model. Our findings of increased serum TNF-α, but not IL-1β, levels in the Chow + 0 mg and HFD + sham mice compared to sham-exposed mice fed a chow diet, suggest that circulating cytokines may play a minor role in the HFD-fed mice exposed to additional e-vapour. We demonstrated previously that alterations in adipose tissue may be one of the major sources of altered systemic immune processes [7]. However, whether the gastrointestinal tract or other organs are also involved in this systemic inflammatory response requires further investigation in future studies.

The lack of effect of e-vapour on the gut microbiome was surprising, especially in light of the discovery that vaping compromises the gut epithelial barrier [18] and changes the oral microbiome in human e-cigarette users [19]. However, our findings also confirm the findings of a human pilot study, where no changes in the gut microbiome were observed [20]. It is important to note that there are no comprehensive human studies assessing the gut microbiome in obese or overweight individuals with e-cigarette use/exposure. Whilst our data provide evidence that e-cigarettes cause no additional changes to the gut microbiome, it should be noted that we adopted a low dose of e-vapour. Therefore, we cannot exclude the possibility that higher doses exposure and the use of different flavours and/or e-cigarette nicotine delivery systems may change the heating temperature and final chemical composition of the heated propylene glycol and/or vegetable glycerine and flavouring compounds and thus affect the gut microbiome profile. Importantly, it is also unknown whether these variables also change the bidirectional gut–lung axis, but it raises important questions for future studies.

It is also important to consider the strain of mice used. In our study, we used Balb/C mice, which is different to previous studies that showed the increased alpha diversity of the gut microbiome in HFD-fed C57Bl/6 or Sv129 mice compared to low-fat diet [21]. This may be due to the difference in strain susceptibility of HFD-induced obesity and related metabolic disorders, whereas these disorders can be less severe in Balb/c mice. There are currently no studies comparing HFD-induced changes in alpha and beta diversity in different strains of mice; however, further research will be required in future studies to decipher potential differences between our current study and previously published studies.

Together, our study highlights both similarities and differences in how the body responds to e-cigarettes/vaping, and it is therefore likely that the long-term sequelae of vaping does not involve the significant alteration of the gut microbiome.

## Figures and Tables

**Table 1:** Body weight and fat mass at 16 weeks.

	Chow + Sham	Chow + E-Cig18	Chow + E-Cig0	HFD + Sham	HFD + E-Cig18	HFD + E-Cig0
**Body weight (g)**	27.6 ± 0.31	25.9 ± 0.72	26.0 ± 0.53	29.7 ± 0.81 *	27.8 ± 0.80 * #	26.7 ± 0.77 #
**Retroperitoneal fat (g)**	0.171 ± 0.025	0.121 ± 0.040	0.125 ± 0.025	0.483 ± 0.103 **	0.263 ± 0.059 ##	0.199 ± 0.022 ##
**Retroperitoneal fat (%)**	0.618 ± 0.090	0.467 ± 0.159	0.490 ± 0.106	1.59 ± 0.296 **	0.94 ± 0.205 #	0.74 ± 0.076 ##
**Epididymal fat (g)**	0.485 ± 0.049	0.422 ± 0.092	0.368 ± 0.007	1.07 ± 0.123 **	0.79 ± 0.090 ** #	0.88 ± 0.113 **
**Epididymal fat (%)**	1.75 ± 0.17	1.62 ± 0.34	1.42 ± 0.048	3.57 ± 0.330 **	2.84 ± 0.230 **	3.27 ± 0.355 **
**Serum triglycerides (mg/mL)**	1.98 ± 0.15	1.74 ± 0.18	1.71 ± 0.19	3.36 ± 0.32 **	2.40 ± 0.37 #	2.41 ± 0.23 #
**Serum NEFA (nM)**	5.63 ± 0.47	3.39 ± 0.35 #	4.36 ± 0.20	8.94 ± 1.04 **	5.72 ± 0.64 * ##	6.38 ± 0.45 * ##

Results are expressed as mean ± S.E.M, *n* = 5–6. HFD effect * *p* < 0.05, ** *p* < 0.01 compared with the chow-fed group with the same treatment; e-vapour effect # *p* < 0.05, ## *p* < 0.01 compared with the Sham-exposed group fed the same diet (two-way ANOVA, Fisher’s least significant difference test). Sham: exposed to ambient air; e-cig18: exposed to nicotine-containing e-vapour; e-cig0: exposed to nicotine-free e-vapour; HFD: high-fat diet; NEFA: non-esterified fatty acids.

## Data Availability

All datasets are available upon request. All codes used in this study are publicly available and referenced in the Materials and Methods section.
